# Practical management of older adults with cancer: geriatric oncology in Japan

**DOI:** 10.1093/jjco/hyac118

**Published:** 2022-07-22

**Authors:** Tomonori Mizutani

**Affiliations:** Department of Medical Oncology, Kyorin University Faculty of Medicine, Japan

**Keywords:** geriatric oncology, older adult, frailty, geriatric assessment, evidence-based medicine

## Abstract

Japan has the highest proportion of older adults globally, and the average life expectancy of the Japanese population has increased in recent decades. Given that the incidence of cancer increases with age, it is a major health concern for older adults. However, geriatric oncology is a relatively new field and collaboration between oncologists and geriatricians in Japan is limited. Hence, oncologists and other healthcare professionals engaged in cancer care have not been able to adequately understand geriatric care, and information and experience are insufficient for this specific population. Thus, they may struggle with the assessment and management of older adults with cancer. Recently, several Japanese academic societies for cancer have developed practical guidelines and research policy with regard to geriatric research in older adults with cancer, in addition to organizing symposia and workshops focusing especially on geriatric oncology. Furthermore, because the Japan Geriatrics Society established a discipline committee on cancer, close collaboration between oncologists and geriatricians has grown steadily. Geriatric oncology is currently recognized as an important field of cancer care in Japan. The integration of oncology and geriatric care is anticipated in the near future. However, understanding the aspects of geriatric care and meanings of technical jargons used in geriatric oncology is difficult. Accordingly, this article provides an overview of the current knowledge and recent advancements in geriatric oncology. In addition, it outlines the current status and problems of geriatric oncology in Japan.

## Teaser

This article provides an overview of the current knowledge and recent advances in geriatric oncology, outlining its current status and problems in Japan.

## Introduction

Japan has the highest proportion of older adults worldwide (1, 2). In 2021, 28.8% of the total population in Japan was aged >65 years and 14.9% were aged >75 years (3,4). The average life expectancy of the Japanese population has increased in recent decades; it is nearly 87.7 years for women and 81.6 years for men. By 2065, every 1 in 2.6 people will be aged ≥65 years and every 1 in 3.9 people will be aged ≥75 years in Japan (5). Given that the incidence of cancer increases with age, >70% of new cancer cases are reported in patients aged ≥65 years in Japan (6, 7). Cancer is the leading cause of death in Japan, followed by heart disease and pneumonia (8). Thus, cancer is a major health concern for older adults in Japan.

To the best of our knowledge, no standardized assessment and management approaches have been established for older adults with cancer. Healthcare providers frequently face several challenges because of various reasons. First, there has been little collaboration between oncologists and geriatricians in Japan; oncologists and other healthcare professionals engaged in cancer care are not sufficiently knowledgeable or experienced in geriatric care for this specific population (9). Second, reliable evidence to support therapeutic decision-making in this population is limited, because older adults with cancer are generally underrepresented in clinical trials, particularly those with comorbidities and those who use concomitant medications (10). Finally, the rapid discovery and development of new approaches in medicine have resulted in ambiguities among oncologists and other healthcare professionals engaged in cancer care. Traditional therapies such as treatment with cytotoxic drugs may be less effective and associated with a greater toxicity in older adults with cancer. However, some novel agents such as molecularly targeted or immunomodulatory agents are more effective and better tolerated in comparison with traditional therapies, even among vulnerable older adults with cancer (11,12,13). Hence, oncologists and other healthcare professionals engaged in cancer care may struggle with the management of older adults with cancer. Hence, establishing a specialized field for older adults is warranted.

Geriatric oncology is a relatively new field that focuses on the assessment and management of older adults with cancer; this field has expanded over the past two decades (14). The American Society of Clinical Oncology (ASCO) (15) and International Society of Geriatric Oncology (SIOG) (16) have been the pioneers in the field of geriatric oncology. These organizations have formulated several guidelines on geriatric oncology to assist healthcare professionals in understanding the assessment and management of older adults with cancer. Although Japan had been behind in the field of geriatric oncology, several Japanese academic societies for cancer-related research and development, such as the Japanese Society of Medical Oncology (JSMO) (17), Japanese Society of Clinical Oncology (JSCO) (18) and Japan Clinical Oncology Group (JCOG) (19), have recently developed practical guidelines and research policy with regard to geriatric research in older adults with cancer, in addition to organizing symposia and workshops focusing especially on geriatric oncology (20). Geriatric oncology has thus been recognized as a key field of cancer care in Japan. However, understanding the aspects of geriatric care and comprehending the meaning of the jargon used in geriatric oncology remain challenging. Therefore, this article provides an overview of the current knowledge and recent advances in geriatric oncology; moreover, it outlines the current status and problems of geriatric oncology in Japan.

## Definition of older adults

There are no concrete definitions of ‘older adults.’ A chronological definition is commonly used, but to the best of our knowledge, there is no universally accepted cutoff age for identifying patients as ‘older adults.’ However, in many countries including Japan, individuals aged >65 years are typically referred to as ‘older adults’ (2,21). The use of chronological age is a simple way to describe senior citizens, particularly at regulatory institutions. However, the existing medical or biological evidence to support this definition remains unclear. Chronological age alone fails to address the heterogeneity in the physiological and functional statuses of older adults (22,23). Thus, factors other than chronological age are needed to clarify this heterogeneity in older adults with cancer.

Frailty is a state of increased vulnerability that increases the risk of adverse health-related outcomes following a stressor event (24,25,26). Although there is no particular definition for frailty (27,28,29,30), previous studies have stated that it is an extreme consequence of the normal aging process; it is a multidimensional state with both physical and psychosocial factors, as well as a dynamic state; i.e. it may be reversed or attenuated by interventions focusing on its underlying causes (31,25). The concept of frailty is being increasingly recognized as a crucial healthcare issue (32,33,34) given its association with increased risks of mortality, hospitalization, falls and admission to long-term care. Frailty may be reversed, i.e. individuals can dynamically transition between severity states through interventions focusing on the underlying causes and identifying their presence of such underlying causes of frailty, such as nutritional deficiency, poor mobility, incontinence and delirium; this may help oncologists and other healthcare professionals engaged in cancer care personalize treatment plans for older adults with cancer (35).

Although frailty is a popular concept that can appropriately describe the heterogeneity in older adults, the comprehension of this concept differs between oncologists and geriatricians (Table 1) (36,37). Geriatricians typically use the term ‘frailty’ to describe a dynamic state of increased vulnerability that increases the risk of adverse health-related outcomes after a stressor and is associated with a higher likelihood of functional decline, disability, hospitalization and mortality. Conversely, geriatric oncologists typically use the term to describe an older adult who is generally unfit to receive cancer treatment and is best suited for supportive care or palliative treatment (38). This inconsistency has led to ambiguities among geriatricians and oncologists as well as other healthcare professionals engaged in cancer care. To the best of our knowledge, there is no robust definition of ‘older adults’ or ‘frailty’ in geriatric oncology, and the meaning of these terms can change depending on the context; thus, the terms must not be used interchangeably and only as per the context.

**Table 1:** Definitions of various terms used by geriatric oncologists and geriatricians

Term	Geriatric oncology	Geriatrics
Frailty	Commonly used: an older individual who is generally unfit for cancer treatment and should receive best-suited supportive care or palliative treatment	Commonly used: A state of increased vulnerability, including an extreme consequence of the normal aging process; it is a multidimensional state with physical and psychosocial factors as well as a dynamic state; i.e. it may be reversed
CGA	Commonly used: often confused with GA or several domains of GA	Commonly used: A multidimensional diagnostic process to identify the care needs older adults with vulnerability, plan their care, and improve their outcomes
GA	Commonly used: diagnostic process that is sometimes not a systematic evaluation	Not used
GA and intervention (GA and management)	Commonly used: evaluation and development of a treatment plan based on GA	Not used
Geriatric screening (abbreviated as CGA, mini-CGA)	Commonly used: any short measure or series of measures designed to identify patients who would benefit from a CGA or several domains of GA	Rarely used: any short measure or series of measures designed to identify patients who would benefit from a CGA

CGA, comprehensive geriatric assessment; GA, geriatric assessment

## Assessment of older adults with cancer

Assessing the health status of older adults with cancer is important to support therapeutic decision-making for this population. Chronological age and performance status (PS) such as the Eastern Cooperative Oncology Group or Karnofsky performance status, cannot address the heterogeneity in older adults with cancer; thus, they are not good indicators of their physiological and functional statuses (39,40).

### Comprehensive geriatric assessment and geriatric assessment

The term ‘comprehensive geriatric assessment (CGA)’ is commonly used in the field of geriatrics and has been defined as a multidimensional and interdisciplinary, diagnostic process to identify the care needs of older adults with vulnerability, plan their care and improve treatment outcomes (41,42,43). The key domains of CGA include physical health (i.e. comorbidities, medication use and nutritional status), functional status (i.e. basic and instrumental activities of daily living), psychological status (i.e. cognitive and emotional status) and socioeconomic factors (i.e. living situation and financial resources) (37). Fundamentally, CGA has six components: (1) data gathering, (2) discussion among the team, (3) development of a treatment plan, (4) implementation of the treatment plan, (5) monitoring treatment response and (6) revising the treatment plan, if needed. All these components indicate that CGA is not only a diagnostic process, but also aids the development and implementation of a treatment plan (44). For instance, after evaluating the key domains, the patients’ preferences and treatment goals should be discussed, for the care plan to reflect these crucial aspects of care (45). In addition, tailored interventions that address the patient’s vulnerability should be subsequently recommended, such as nutritional supplements or home nursing to help with medications (46,47,42). Monitoring and replanning are also essential (48).

The term ‘geriatric assessment (GA)’ is specifically used in the field of geriatric oncology. Most clinical studies on CGA in the field of oncology have focused on gathering data but not on providing tailored care based on CGA findings. Because of this, the SIOG recommends using the term ‘GA’ rather than ‘CGA’ (49). Thus, GA is generally recognized as a diagnostic process for evaluating the physical health, functional status, psychological status and socioeconomic factors for older adults with cancer (49,37) (Table 1). However, lack of clarity among geriatricians and oncologists may be attributable to the fact that GA is performed regardless of whether all or some of the aforementioned domains are evaluated. Identifying the domains evaluated is essential. Nevertheless, GA is considered to be valuable in oncology practice owing to its abilities to detect an impairment not identified in the routine history or physical examination, predict severe treatment-induced toxicity, and predict overall survival in various tumours and treatment settings (50). In addition, GA can guide decision-making via discussion among oncologists and other healthcare professionals engaged in cancer care (51). Thus, several academic societies such as the ASCO, SIOG, European Society for Medical Oncology (ESMO) (52), Cancer and Aging Research Group (CARG) (53) and National Comprehensive Cancer Network (54) have recommended GA for use in clinical practice and research.

As mentioned previously, CGA is performed for both the evaluation and implantation of interventions to improve the outcomes of older adults; conversely, GA is performed only for diagnostic purposes among older adults with cancer. Recently, several studies have reported that GA and interventions focused on the vulnerability of older adults may improve treatment outcomes, resulting in low chemotherapy-induced toxicity, low rate of postoperative complications, and improved functional status and quality of life (QOL) (38,51,55–57,58, 59). GA outcomes and required interventions can enable the development of integrated and individualized plans for the management of patients with cancer and identification of nononcologic concerns that are amenable to intervention (Table 1).

### Geriatric screening

The term ‘geriatric screening’ is also used in the field of oncology. It refers to the use of one or more short screening tools to identify older adults with cancer who may benefit from CGA (60,61) (Table 1). Despite accumulating evidence suggesting the importance of GA in geriatric oncology, the lack of time and resources prevents the routine implementation of GA in oncological practice (62). Geriatric screening may be less burdening in terms of time and resources than CGA or several domains of GA in older adults with cancer; however, GA cannot replace CGA. Geriatric screening helps identify patients at the highest risk of poor outcomes following cancer treatment and devise better treatment strategies for them (63). Among the available geriatric screening tools, G8—the Triage Risk Screening Tool—and Vulnerable Elders Survey-13 are supported with the highest amount of evidence with regard to their use in clinical practice (64,65). To the best of our knowledge, no single tool or approach has been recommended for this specific population. However, any tool is encouraged as the initial step to aid clinicians in identifying the at-risk older adults with cancer before treatment initiation. The ASCO guidelines encourage clinicians to routinely incorporate geriatric measures to assess baseline function and other geriatric domains in older adults with cancer who are undergoing or considering chemotherapy (43).

### Risk prediction models for chemotherapy

Models predicting the risk of severe chemotherapy-induced toxicity may aid treatment decisions for older adults with cancer and can be made after considering the balance between the benefits and drawbacks of cancer treatment. The CARG toxicity score and Chemotherapy Risk Assessment Scale for High-Age Patients (CRASH) score are widely used for predicting severe chemotherapy-induced toxicity in older adults with cancer (66–68,69). Both scores are recommended for assessing the risk of chemotherapy-induced toxicity.

**Figure 1:**
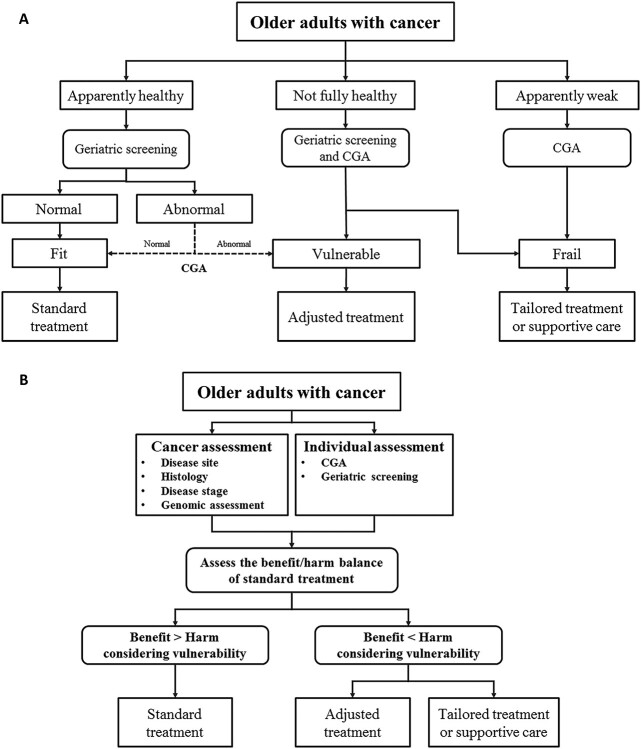
Assessment schemes for older adults with cancer. (a) Treatment strategies based on vulnerability. (b) Treatment strategies based on the benefit/harm balance and vulnerability. CGA, comprehensive geriatric assessment.

## Management of older adults with caner

### General approach for older adults with caner

After assessment of the patient, a treatment strategy is selected. The most frequently used approach for developing treatment strategies in the field of geriatric oncology is categorizing older adults with cancer into three groups based on their vulnerability: fit, vulnerable, and frail (14,70) (Fig. 1a). Fit patients may benefit from standard cancer treatment similarly as younger patients, vulnerable patients may benefit from adjusted therapy, and frail patients may benefit from best-suited supportive care or palliative treatment. Given the simplicity of this approach and that it is easy to understand, it has gained popularity. However, it has resulted in ambiguity among geriatricians and oncologists for various reasons. First, the term ‘frail’ has distinctly different meanings in the fields of geriatrics and oncology (Table 1). Second, to the best of our knowledge there is no standard method for categorizing older adults with cancer using the aforementioned classification (52). Although some classification strategies have been postulated based on GA or geriatric screening, these classifications were inconsistent and based only on clinical expertise and consensus (35). Third, this approach was developed >20 years ago; it does not reflect the advancements in medicine, such as the development of molecularly targeted or immunomodulatory agents with greater efficacy and less toxicity than cytotoxic drugs.

Another approach for developing treatment strategies is based on the consideration of both patients’ vulnerability and the benefit/harm balance of cancer treatments (71) (Fig. 1b). This approach reflects the advancements in medicine, which means that treatment-induced toxicity can be changed based on the characteristics of each type of cancer treatment. In addition, this approach is flexible according to the condition of older adults with cancer. The fit, vulnerable, and frail classification is generally based on patients’ vulnerability before receiving treatment, but the vulnerability of older adults is based on their condition throughout the treatment period. The approach of benefit/harm balance allows treatment intensity to be adjusted according to the patients’ vulnerability at any point during the treatment course.

### Practical approaches for older adults with cancer

**Figure 2:**
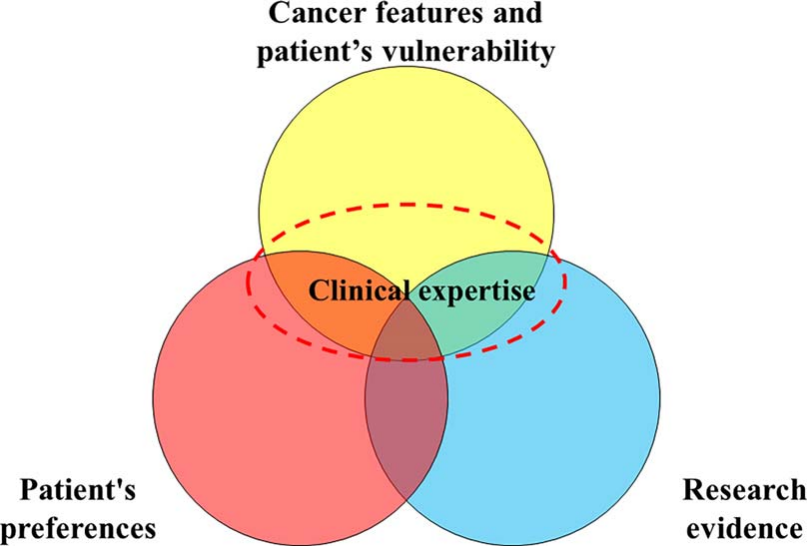
Evidence-based medicine for older adults with cancer.

In daily medical practice, making treatment decisions for older adults with cancer is complex because oncologists and other healthcare professionals engaged in cancer care must consider substantial information about not only cancer but also their patients, such as the patients’ vulnerability, preferences and circumstances. This practical approach is an actual evidence-based medicine (EBM) approach (72,73). In the EBM approach, treatment decisions must consider the following: (1) patients’ clinical and physical circumstances, (2) research evidence, (3) patients’ preferences and (4) critical appraisal with clinical expertise. This approach is useful for routine medical practice of oncologists and other healthcare professionals engaged in cancer care (Fig. 2).

#### Patient’s clinical and physical circumstances

The first step is to understand the clinical status of both the cancer and patients. Treatment decisions in older adults with cancer should be made considering the abovementioned clinical and physical circumstances, to determine available treatment options. The clinical status of cancers includes disease site, histology, disease stage, genomic assessment, disease prognosis and treatment-related benefits and drawbacks; conversely, patient status includes their vulnerabilities, such as physical health, functional status, psychological status and socioeconomic factors other than PS. CGA or GA may help clarify the heterogeneity in older adults with cancer. Moreover, the field of genomics is developing rapidly; understanding the molecular biology of cancer is becoming increasingly important, even in geriatric oncology. In the era of precision medicine for older adults with cancer, the concept of precision medicine should extend beyond the use of tumour-specific markers to incorporate the evaluation of the health status of older adults with cancer; the latter is assessed as part of routine GA and has been demonstrated to significantly affect patient outcomes (74).

#### Research evidence

The second step is to identify and critically appraise the best evidence. However, there are very few reliable studies that support therapeutic decision-making for older adults with cancer, a population that is generally underrepresented in clinical trials, particularly those with severe comorbidities or functional dependencies (75,76,77). In addition, if the enrolled patients are healthier than the general population of older adults with cancer, the results of the trials may not be generalizable to real-world situations. Thus, it is important to carefully interpret the results of clinical trials for older adults with cancer (78,79).

#### Patient preferences

The third step is to consider the patients’ preferences. Recently, patient preferences were incorporated into the first model of EBM; the importance of these preferences was emphasized in a revised version (73). It is important to determine the preferences of older adults with cancer, because intensive treatment may result in greater all-cause mortality in this population, although the practice of oncology often focuses on survival-related outcomes (80,81,82). Oncologists and other cancer healthcare professionals engaged in cancer care should ensure that the patient’s own priorities are congruent with the provider’s treatment goals in the case of advanced disease (83,84,85,86). A Japanese study demonstrated a discrepancy in the prioritization of healthcare outcomes between healthcare professionals and older patients and their families (87). Healthcare professionals considered ‘improvement of QOL’ to be the most important goal, but older patients and their families considered this less important than the ‘effective treatment of illness’ and ‘improvement of physical function.’ Thus, it is important to discuss treatment goals with patients and their families, making efforts to involve patients in treatment decisions and consider their preferences whenever possible.

#### Critical appraisal with clinical expertise

The last step is to integrate these considerations into clinical expertise. Critical appraisal with clinical expertise is needed to integrate patients’ clinical and physical circumstances, research evidence and patients’ preferences. Shared decision-making is important, particularly for older adults with cancer, to recommend the most appropriate treatment that is neither overtreatment nor undertreatment (88).

### Practical approach for older adults with cancer and dementia

Cognitive impairment is highly prevalent among older adults, which hampers their decision-making capacity. In addition, brain metastases occur in approximately 25% of adults with cancer, with nearly 50% of such events impairing patients’ understanding of medical decisions (89). Although discussions with patients regarding the treatment goals are important, this is sometimes difficult (90). However, this does not mean that treatment goals should be decided without any input from the patients (91). Patients with cognitive impairment do not always possess decision-making capacity, the Japanese government published a guideline for healthcare professionals to support decision-making when treating patients with cognitive impairment.

Advance care planning (ACP) enables individuals to define goals and preferences for future medical treatment and care, discuss these goals and preferences with their families and healthcare providers, and record and review them, if appropriate (92,93,94,95, 96). Furthermore, the Japanese government has published guidelines to select the most suitable and best decision support model and encouraged the promotion of ACP.

## Current situation and future prospects of geriatric oncology in Japan

Geriatric oncology is an emerging field that is undergoing continuous development worldwide. In 2021, the SIOG updated their list of priorities for improving the care of older adults with cancer worldwide with the goal of incorporating various data into clinical practice (97). The present list includes four priority domains: (1) education, (2) clinical practice, (3) research and (4) strengthening collaborations and partnerships. It is important to evaluate geriatric oncology in Japan according to this list. With regard to education, several academic societies for cancer have provided educational materials and held conferences focused on geriatric oncology (17,18). Although the SIOG policy review recommends integrating geriatric oncology into educational programs in medical, nursing and allied health profession schools, in addition to educating the general public about the relevance of providing age-appropriate care for older adults with cancer, efforts have been insufficient in Japan. The next step is to develop an educational system to address these issues.

With regard to clinical practice, the JSMO and JSCO published a clinical practical guideline for older adults with cancer that presents recommended treatment options for older adults with several types of cancer (17). Given few clinical practice guidelines for older adults with cancer worldwide, these guidelines may be useful for Japanese clinicians. The SIOG policy review recommended establishing centres of excellence in geriatric oncology for providing clinical care. ESMO has launched multiple programs for cancer centres that provide highly advanced and integrated oncology and palliative care services. The next step is to integrate geriatrics with oncology and palliative care. The SIOG policy review further recommends developing and implementing models to provide optimal care for older adults with cancer. The Japanese government has attempted to establish ‘a community-based integrated care system’; this system includes healthcare professionals who fully understand the physical and mental characteristics of older adults (98). This system is intended for older adults without cancer, but it may also help older adults with cancer and their families.

With regard to research, the JCOG has developed a geriatric research policy, establishing the standard endpoints and methodological schemes for geriatric research (10). Japanese researchers have published several important studies on older adults with cancer (99,100,101,102, 103,104,105). However, methodological schemes for geriatric research are rarely designed for older adults. Instead, the methodological schemes developed for younger patients are usually applied to older adults. For instance, the primary endpoints of clinical trials conducted in adults are usually survival-related outcomes such as overall survival, which may not reflect the true benefit for older adults with cancer (106,107,108). A more optimal method for older adults with cancer is warranted, for instance, to allow the evaluation of treatment outcomes among other aspects.

The SIOG policy review recommended strengthening collaborations and partnerships with other fields. Traditionally, geriatricians have treated older adults ‘without’ cancer and oncologists have treated older adults ‘with’ cancer, which resulted in poor collaboration between these professionals. Recently, as the Japan Geriatrics Society has established a discipline committee on cancer (109), close collaboration between oncologists and geriatricians may be possible in the near future.

Japan’s demographics have substantially changed in recent years, with substantial reductions in fertility rates and considerable increase in life expectancy (5, 4). These factors have increased the cost of maintaining the healthcare system and the burden on families (110). Japan’s Healthcare Insurance System ensures that anyone can receive necessary medical treatment (111,112), and it has a system called ‘high-cost medical expense benefit,’ wherein patients are required to pay a fixed ceiling amount despite extremely high medical costs. Because the government allows all patients with universal health insurance to use expensive drugs, most clinicians prescribe such drugs to all patients, even the centenarians with vulnerabilities. The medical costs for treating individuals aged ≥75 years in the fiscal year 2019 comprised approximately 37% of the national medical care expenditure, reflecting a slight upward trend. The Japanese government must devise a new plan which maintains the current standard of healthcare facilities, while prolonging the life expectancy and quality of older adults with cancer.

## Conclusions

The field of geriatric oncology is undoubtedly developing in Japan; however, several challenges remain with regard to the care of older adults with cancer. However, because the collaboration between oncologists and geriatricians has grown steadily, the integration of oncology and geriatric care is anticipated in the near future.

## Funding

This work was supported by JSPS KAKENHI grant number JP20K18860.

## Conflict of interest statement

None declared.

## References

[1] World Health Organization . World Health Statistics. 2021;2021. https://apps.who.int/iris/bitstream/handle/10665/342703/9789240027053-eng.pdf.

[2] World Health Organization . World report on ageing and health. Geneva: World Health Organization, 2015; 2015.

[3] Statistics Bureau of Japan . Statistical Handbook of Japan 2021. https://www.stat.go.jp/english/data/handbook/index.html.

[4] National Institute of Population and Social Security Research . Population and social security in Japan 2019. https://www.ipss.go.jp/s-info/e/pssj/pssj2019.pdf.

[5] Muramatsu N, Akiyama H. Japan: super-aging society preparing for the future. Gerontologist 2011;51(4):425–32. 21804114 10.1093/geront/gnr067

[6] Foundation for Promotion of Cancer Research . Cancer STATISTICS in Japan 2021. https://ganjoho.jp/public/qa_links/report/statistics/2021_en.html.

[7] Cinar D, Tas D. Cancer in the elderly. Northern clinics of Istanbul 2015;2(1):73–80. 28058345 10.14744/nci.2015.72691 PMC5175057

[8] Yang L, Fujimoto J, Qiu D, Sakamoto N. Trends in cancer mortality in the elderly in Japan, 1970-2007. Annals of oncology : official journal of the European Society for Medical Oncology. 2010;21(2):389–96. 19622594 10.1093/annonc/mdp303

[9] Kadambi S, Loh KP, Dunne R, Magnuson A, Maggiore R, Zittel J, et al. Older adults with cancer and their caregivers - current landscape and future directions for clinical care. Nat Rev Clin Oncol 2020;17(12):742–55. 32879429 10.1038/s41571-020-0421-z PMC7851836

[10] Mizutani T, Nakamura K, Fukuda H, Ogawa A, Hamaguchi T, Nagashima F. Geriatric research policy: Japan clinical oncology group (JCOG) policy. Jpn J Clin Oncol 2019;49(10):901–10. 31565730 10.1093/jjco/hyz093 PMC6886463

[11] Nosaki K, Saka H, Hosomi Y, Baas P, de Castro G Jr, Reck M, et al. Safety and efficacy of pembrolizumab monotherapy in elderly patients with PD-L1-positive advanced non-small-cell lung cancer: pooled analysis from the KEYNOTE-010, KEYNOTE-024, and KEYNOTE-042 studies. Lung Cancer (Amsterdam, Netherlands) 2019;135:188–95. 10.1016/j.lungcan.2019.07.004 31446994

[12] Daste A, Chakiba C, Domblides C, Gross-Goupil M, Quivy A, Ravaud A, et al. Targeted therapy and elderly people: a review. *Eur J Cancer* (Oxford, England 1990) 2016;69:199–215. 10.1016/j.ejca.2016.10.005 27855351

[13] Elias R, Morales J, Presley C. Checkpoint inhibitors for non-small cell lung cancer among older adults. Curr Oncol Rep 2017;19(9):62. 28755314 10.1007/s11912-017-0619-0

[14] Balducci L. Geriatric oncology. Crit Rev Oncol Hematol 2003;46(3):211–20. 12791420 10.1016/s1040-8428(03)00020-9

[15] American Society of Clinical Oncology https://www.asco.org/.

[16] International Society of Geriatric Oncology https://www.siog.org/.

[17] Japanese Society of Medical Oncology https://www.jsmo.or.jp/en/.

[18] Japanese Society of Clinical Oncology http://www.jsco.or.jp/english/.

[19] The Japan Clinical Oncology Group http://www.jcog.jp/en/.

[20] JCOG. Geriatric Study Committee . http://www.jcog.jp/en/committee/index.html

[21] World Health Organization . Global strategy and action plan on ageing and health. http://wwwwhoint/ageing/WHO-GSAP-2017pdf?ua=1 (1 February 2018).

[22] Ouchi Y, Rakugi H, Arai H, Akishita M, Ito H, Toba K, et al. Redefining the elderly as aged 75 years and older: proposal from the joint Committee of Japan Gerontological Society and the Japan geriatrics society. Geriatr Gerontol Int 2017;17(7):1045–7. 28670849 10.1111/ggi.13118

[23] Soto-Perez-de-Celis E, Li D, Yuan Y, Lau YM, Hurria A. Functional versus chronological age: geriatric assessments to guide decision making in older patients with cancer. Lancet Oncol 2018;19(6):e305-e16. 29893262 10.1016/S1470-2045(18)30348-6

[24] Chan MLT, Yu DSF. The effects of low-impact moderate-intensity stepping exercise on fatigue and other functional outcomes in older adults with multimorbidity: a randomized controlled trial. Arch Gerontol Geriatr 2022;98:104577. 34808440 10.1016/j.archger.2021.104577

[25] Dent E, Martin FC, Bergman H, Woo J, Romero-Ortuno R, Walston JD. Management of frailty: opportunities, challenges, and future directions. Lancet (London, England). 2019;394(10206):1376–86. 10.1016/S0140-6736(19)31785-4 31609229

[26] The IAGG and its GARN Networ . White book on frailty 2016 https://www.jpn-geriat-soc.or.jp/gakujutsu/pdf/whitebook.pdf.

[27] Fried LP, Tangen CM, Walston J, Newman AB, Hirsch C, Gottdiener J, et al. Frailty in older adults: evidence for a phenotype. J Gerontol A Biol Sci Med Sci 2001;56(3):M146–56. 11253156 10.1093/gerona/56.3.m146

[28] Satake S, Arai H. The revised Japanese version of the cardiovascular health study criteria (revised J-CHS criteria). Geriatr Gerontol Int 2020;20(10):992–3. 33003255 10.1111/ggi.14005

[29] Rockwood K, Mitnitski A. Frailty defined by deficit accumulation and geriatric medicine defined by frailty. Clin Geriatr Med 2011;27(1):17–26. 21093719 10.1016/j.cger.2010.08.008

[30] Rockwood K, Song X, MacKnight C, Bergman H, Hogan DB, McDowell I, et al. A global clinical measure of fitness and frailty in elderly people. CMAJ : Canadian Medical Association Journal = journal de l'Association medicale canadienne 2005;173(5):489–95. 10.1503/cmaj.050051 PMC1188185 16129869

[31] Hoogendijk EO, Afilalo J, Ensrud KE, Kowal P, Onder G, Fried LP. Frailty: implications for clinical practice and public health. Lancet (London, England) 2019;394(10206):1365–75. 10.1016/S0140-6736(19)31786-6 31609228

[32] Kojima G. Frailty as a predictor of disabilities among community-dwelling older people: a systematic review and meta-analysis. Disabil Rehabil 2017;39(19):1897–908. 27558741 10.1080/09638288.2016.1212282

[33] Ensrud KE, Ewing SK, Taylor BC, Fink HA, Stone KL, Cauley JA, et al. Frailty and risk of falls, fracture, and mortality in older women: the study of osteoporotic fractures. J Gerontol A Biol Sci Med Sci 2007;62(7):744–51. 17634322 10.1093/gerona/62.7.744

[34] Soysal P, Veronese N, Thompson T, Kahl KG, Fernandes BS, Prina AM, et al. Relationship between depression and frailty in older adults: a systematic review and meta-analysis. Ageing Res Rev 2017;36:78–87. 28366616 10.1016/j.arr.2017.03.005

[35] Ferrat E, Paillaud E, Caillet P, Laurent M, Tournigand C, Lagrange JL, et al. Performance of four frailty classifications in older patients with cancer: prospective elderly cancer patients cohort study. J Clin Oncol 2017;35(7):766–77. 28095145 10.1200/JCO.2016.69.3143 PMC6366272

[36] Hurria A, Dale W, Mooney M, Rowland JH, Ballman KV, Cohen HJ, et al. Designing therapeutic clinical trials for older and frail adults with cancer: U13 conference recommendations. J Clin Oncol 2014;32(24):2587–94. 25071116 10.1200/JCO.2013.55.0418 PMC4129504

[37] Puts MTE, Alibhai SMH. Fighting back against the dilution of the comprehensive geriatric assessment. Journal of Geriatric Oncology 2018;9(1):3–5. 28867559 10.1016/j.jgo.2017.08.009

[38] Puts MT, Hardt J, Monette J, Girre V, Springall E, Alibhai SM. Use of geriatric assessment for older adults in the oncology setting: a systematic review. J Natl Cancer Inst 2012;104(15):1133–63. 22851269 10.1093/jnci/djs285 PMC3413614

[39] Hurria A, Gupta S, Zauderer M, Zuckerman EL, Cohen HJ, Muss H, et al. Developing a cancer-specific geriatric assessment: a feasibility study. Cancer 2005;104(9):1998–2005. 16206252 10.1002/cncr.21422

[40] Sørensen JB, Klee M, Palshof T, Hansen HH. Performance status assessment in cancer patients. An inter-observer variability study. Br J Cancer 1993;67(4):773–5. 8471434 10.1038/bjc.1993.140 PMC1968363

[41] Stuck AE, Siu AL, Wieland GD, Adams J, Rubenstein LZ. Comprehensive geriatric assessment: a meta-analysis of controlled trials. Lancet (London, England). 1993;342(8878):1032–6. 10.1016/0140-6736(93)92884-v 8105269

[42] Reuben DB, Borok GM, Wolde-Tsadik G, Ershoff DH, Fishman LK, Ambrosini VL, et al. A randomized trial of comprehensive geriatric assessment in the care of hospitalized patients. N Engl J Med 1995;332(20):1345–50. 7715645 10.1056/NEJM199505183322007

[43] Mohile SG, Dale W, Somerfield MR, Schonberg MA, Boyd CM, Burhenn PS, et al. Practical assessment and Management of Vulnerabilities in older patients receiving chemotherapy: ASCO guideline for geriatric oncology. J Clin Oncol 2018;36(22):2326–47. 29782209 10.1200/JCO.2018.78.8687 PMC6063790

[44] Reuben DB, Fishman LK, McNabney M, Wolde-Tsadik G. Looking inside the black box of comprehensive geriatric assessment: a classification system for problems, recommendations, and implementation strategies. J Am Geriatr Soc 1996;44(7):835–8. 8675935 10.1111/j.1532-5415.1996.tb03744.x

[45] Overcash J, Ford N, Kress E, Ubbing C, Williams N. Comprehensive geriatric assessment as a versatile tool to enhance the Care of the Older Person Diagnosed with cancer. Geriatrics (Basel) 2019;4(2):1–13. 10.3390/geriatrics4020039 PMC6630523 31238518

[46] Arends J, Bachmann P, Baracos V, Barthelemy N, Bertz H, Bozzetti F, et al. ESPEN guidelines on nutrition in cancer patients. Clinical Nutrition (Edinburgh, Scotland) 2017;36(1):11–48. 10.1016/j.clnu.2016.07.015 27637832

[47] Bozzetti F. Evidence-based nutritional support of the elderly cancer patient. Nutrition (Burbank, Los Angeles County, Calif) 2015;31(4):585–6. 10.1016/j.nut.2014.11.004 25770321

[48] Rubenstein L. The clinical effectiveness of multidimensional geriatric assessment. J Am Geriatr Soc 1983;31(12):758–62. 6655177 10.1111/j.1532-5415.1983.tb03395.x

[49] Wildiers H, Heeren P, Puts M, Topinkova E, Janssen-Heijnen ML, Extermann M, et al. International Society of Geriatric Oncology consensus on geriatric assessment in older patients with cancer. J Clin Oncol 2014;32(24):2595–603. 25071125 10.1200/JCO.2013.54.8347 PMC4876338

[50] Corre R, Greillier L, Le Caër H, Audigier-Valette C, Baize N, Bérard H, et al. Use of a comprehensive geriatric assessment for the Management of Elderly Patients with advanced non-small-cell lung Cancer: the phase III randomized ESOGIA-GFPC-GECP 08-02 study. J Clin Oncol 2016;34(13):1476–83. 26884557 10.1200/JCO.2015.63.5839

[51] Mohile SG, Epstein RM, Hurria A, Heckler CE, Canin B, Culakova E, et al. Communication with older patients with cancer using geriatric assessment: a cluster-randomized clinical trial from the National Cancer Institute Community oncology research program. JAMA Oncol 2020;6(2):196–204. 31697365 10.1001/jamaoncol.2019.4728 PMC6865234

[52] European Society for Medical Oncology . ESMO HANDBOOK of cancer in the senior patient https://oncologypro.esmo.org/education-library/handbooks/cancer-in-the-senior-patient.

[53] Cancer and Aging Research Group https://www.mycarg.org/.

[54] National Comprehensive Cancer Network . NCCN GUIDELINES FOR SPECIFIC POPULATIONS: older adult oncology https://www.nccn.org/professionals/physician_gls/default.aspx.

[55] Li D, Sun CL, Kim H, Soto-Perez-de-Celis E, Chung V, Koczywas M, et al. Geriatric assessment-driven intervention (GAIN) on chemotherapy-related toxic effects in older adults with cancer: a randomized clinical trial. JAMA Oncol 2021;7(11):e214158. 34591080 10.1001/jamaoncol.2021.4158 PMC8485211

[56] Mohile SG, Mohamed MR, Xu H, Culakova E, Loh KP, Magnuson A, et al. Evaluation of geriatric assessment and management on the toxic effects of cancer treatment (GAP70+): a cluster-randomised study. Lancet (London, England) 2021;398(10314):1894–904. 10.1016/S0140-6736(21)01789-X PMC8647163 34741815

[57] Mohile SG, Epstein RM, Hurria A, Heckler CE, Canin B, Culakova E, et al. Communication with older patients with cancer using geriatric assessment: a cluster-randomized clinical trial from the National Cancer Institute Community oncology research program. JAMA Oncol 2019;6(2):1–9. 10.1001/jamaoncol.2019.4728 PMC6865234 31697365

[58] Soo W-K, King M, Pope A, Parente P, Darzins P, Davis ID. Integrated geriatric assessment and treatment (INTEGERATE) in older people with cancer planned for systemic anticancer therapy. J Clin Oncol 2020;38(15_suppl):12011. 10.1016/S2666-7568(22)00169-6 36102776

[59] Rostoft S, O'Donovan A, Soubeyran P, Alibhai SMH, Hamaker ME. Geriatric assessment and Management in Cancer. J Clin Oncol 2021;39(19):2058–67. 34043439 10.1200/JCO.21.00089

[60] Decoster L, Van Puyvelde K, Mohile S, Wedding U, Basso U, Colloca G, et al. Screening tools for multidimensional health problems warranting a geriatric assessment in older cancer patients: an update on SIOG recommendationsdagger. Annals of Oncology : Official Journal of the European Society for Medical Oncology. 2015;26(2):288–300. 24936581 10.1093/annonc/mdu210

[61] Garcia MV, Agar MR, Soo WK, To T, Phillips JL. Screening tools for identifying older adults with cancer who may benefit from a geriatric assessment: a systematic review. JAMA Oncol 2021;7(4):616–627. 10.1001/jamaoncol.2020.6736 33443547

[62] Hamaker ME, Wildes TM, Rostoft S. Time to stop saying geriatric assessment is too time consuming. J Clin Oncol Off J Am Soc Clin Oncol 2017;35(25):2871–4. 10.1200/JCO.2017.72.8170 28628364

[63] Hamaker ME, Jonker JM, de Rooij SE, Vos AG, Smorenburg CH, van Munster BC. Frailty screening methods for predicting outcome of a comprehensive geriatric assessment in elderly patients with cancer: a systematic review. Lancet Oncol 2012;13(10):e437–44. 23026829 10.1016/S1470-2045(12)70259-0

[64] Kenis C, Decoster L, Van Puyvelde K, De Grève J, Conings G, Milisen K, et al. Performance of two geriatric screening tools in older patients with cancer. J Clin Oncol Off J Am Soc Clin Oncol 2014;32(1):19–26. 10.1200/JCO.2013.51.1345 24276775

[65] Kenis C, Bron D, Libert Y, Decoster L, Van Puyvelde K, Scalliet P, et al. Relevance of a systematic geriatric screening and assessment in older patients with cancer: results of a prospective multicentric study. Annals of Oncology: Official Journal of the European Society for Medical Oncology 2013;24(5):1306–12. 23293115 10.1093/annonc/mds619

[66] Hurria A, Togawa K, Mohile SG, Owusu C, Klepin HD, Gross CP, et al. Predicting chemotherapy toxicity in older adults with cancer: a prospective multicenter study. J Clin Oncol Off J Am Soc Clin Oncol 2011;29(25):3457–65. 10.1200/JCO.2011.34.7625 PMC3624700 21810685

[67] Hurria A, Mohile S, Gajra A, Klepin H, Muss H, Chapman A, et al. Validation of a prediction tool for chemotherapy toxicity in older adults with cancer. J Clin Oncol Off J Am Soc Clin Oncol 2016;34(20):2366–71. 10.1200/JCO.2015.65.4327 PMC5321104 27185838

[68] Extermann M, Chen H, Cantor AB, Corcoran MB, Meyer J, Grendys E, et al. Predictors of tolerance to chemotherapy in older cancer patients: a prospective pilot study. Eur J Cancer (Oxford, England : 1990). 2002;38(11):1466–73. 12110492 10.1016/s0959-8049(02)00090-4

[69] Extermann M, Boler I, Reich RR, Lyman GH, Brown RH, DeFelice J, et al. Predicting the risk of chemotherapy toxicity in older patients: the chemotherapy risk assessment scale for high-age patients (CRASH) score. Cancer 2012;118(13):3377–86. 22072065 10.1002/cncr.26646

[70] Balducci L, Extermann M. Management of cancer in the older person: a practical approach. Oncologist 2000;5(3):224–37. 10884501 10.1634/theoncologist.5-3-224

[71] DuMontier C, Loh KP, Bain PA, Silliman RA, Hshieh T, Abel GA, et al. Defining undertreatment and overtreatment in older adults with cancer: a scoping literature review. Journal of Clinical Oncology: Official Journal of the American Society of *Clin Oncol* 2020;38(22):2558–2569. 10.1200/JCO.19.02809 PMC7392742 32250717

[72] Sackett DL, Rosenberg WM, Gray JA, Haynes RB, Richardson WS. Evidence based medicine: what it is and what it isn't. BMJ (Clinical research ed). 1996;312(7023):71–2. 10.1136/bmj.312.7023.71 PMC2349778 8555924

[73] Haynes RB, Devereaux PJ, Guyatt GH. Physicians' and patients' choices in evidence based practice. BMJ (Clinical Research ed) 2002;324(7350):1350. 10.1136/bmj.324.7350.1350 PMC1123314 12052789

[74] Williams GR . Geriatric assessment: precision medicine for older adults with cancer. J Oncol Pract 2018;14(2):97–8. 29436301 10.1200/JOP.18.00010

[75] Habr D, McRoy L, Papadimitrakopoulou VA. Age is just a number: considerations for older adults in cancer clinical trials. J Natl Cancer Inst 2021;113(11):1460–4. 33881547 10.1093/jnci/djab070 PMC8562957

[76] Dunn C, Wilson A, Sitas F. Older cancer patients in cancer clinical trials are underrepresented. Systematic literature review of almost 5000 meta- and pooled analyses of phase III randomized trials of survival from breast, prostate and lung cancer. Cancer Epidemiol 2017;51:113–7. 29126091 10.1016/j.canep.2017.11.002

[77] Food and Drug Administration . Inclusion of older adults in cancer clinical trials 2022 https://www.fda.gov/media/156616/download.

[78] Quoix E, Zalcman G, Oster JP, Westeel V, Pichon E, Lavole A, et al. Carboplatin and weekly paclitaxel doublet chemotherapy compared with monotherapy in elderly patients with advanced non-small-cell lung cancer: IFCT-0501 randomised, phase 3 trial. Lancet (London, England). 2011;378(9796):1079–88. 10.1016/S0140-6736(11)60780-0 21831418

[79] Visvanathan K, Levit LA, Raghavan D, Hudis CA, Wong S, Dueck A, et al. Untapped potential of observational research to inform clinical decision making. American Society of Clinical Oncology Research Statement: *J Clin Oncol* 2017;35(16):1845–54. 10.1200/JCO.2017.72.6414 28358653

[80] Sessums LL, Zembrzuska H, Jackson JL. Does this patient have medical decision-making capacity? JAMA 2011;306(4):420–7. 21791691 10.1001/jama.2011.1023

[81] Smith GL, Smith BD. Radiation treatment in older patients: a framework for clinical decision making. J Clin Oncol 2014;32(24):2669–78. 25071132 10.1200/JCO.2014.55.1168 PMC4876341

[82] Fried TR, Bradley EH, Towle VR, Allore H. Understanding the treatment preferences of seriously ill patients. N Engl J Med 2002;346(14):1061–6. 11932474 10.1056/NEJMsa012528

[83] Loh KP, Seplaki CL, Sanapala C, Yousefi-Nooraie R, Lund JL, Epstein RM, et al. Association of prognostic understanding with health care use among older adults with advanced Cancer: a secondary analysis of a cluster randomized clinical trial. JAMA Netw Open 2022;5(2):e220018. 35179585 10.1001/jamanetworkopen.2022.0018 PMC8857680

[84] Stegmann ME, Festen S, Brandenbarg D, Schuling J, van Leeuwen B, de Graeff P, et al. Using the outcome prioritization tool (OPT) to assess the preferences of older patients in clinical decision-making: a review. Maturitas 2019;128:49–52. 31561823 10.1016/j.maturitas.2019.07.022

[85] Yellen SB, Cella DF, Leslie WT. Age and clinical decision making in oncology patients. J Natl Cancer Inst 1994;86(23):1766–70. 7966414 10.1093/jnci/86.23.1766

[86] Fukui S, Yoshiuchi K, Fujita J, Sawai M, Watanabe M. Japanese people's preference for place of end-of-life care and death: a population-based nationwide survey. J Pain Symptom Manage 2011;42(6):882–92. 21689901 10.1016/j.jpainsymman.2011.02.024

[87] Akishita M, Ishii S, Kojima T, Kozaki K, Kuzuya M, Arai H, et al. Priorities of health care outcomes for the elderly. J Am Med Dir Assoc 2013;14(7):479–84. 23415841 10.1016/j.jamda.2013.01.009

[88] Haynes RB, Devereaux PJ, Guyatt GH. Clinical expertise in the era of evidence-based medicine and patient choice. Vox Sang 2002;83 Suppl 1:383–6. 12749371

[89] Nakahori N, Sekine M, Yamada M, Tatsuse T, Kido H, Suzuki M. Future projections of the prevalence of dementia in Japan: results from the Toyama dementia survey. BMC Geriatr 2021;21(1):602. 34702187 10.1186/s12877-021-02540-z PMC8546941

[90] McKoy JM, Burhenn PS, Browner IS, Loeser KL, Tulas KM, Oden MR, et al. Assessing cognitive function and capacity in older adults with cancer. Journal of the National Comprehensive Cancer Network : JNCCN 2014;12(1):138–44. 24453297 10.6004/jnccn.2014.0011

[91] Mitnitski AB, Mogilner AJ, Rockwood K. Accumulation of deficits as a proxy measure of aging. TheScientificWorldJournal 2001;1:323–36. 10.1100/tsw.2001.58 PMC6084020 12806071

[92] Chikada A, Takenouchi S, Nin K, Mori M. Definition and recommended cultural considerations for advance care planning in Japan: a systematic review. Asia Pac J Oncol Nurs 2021;8(6):628–38. 34790847 10.4103/apjon.apjon-2137 PMC8522591

[93] Okada H, Kiuchi T, Okuhara T, Kizawa Y. Effect of advance care planning discussions with trained nurses in older adults with chronic diseases in Japan. Annals of Palliative Medicine 2021;11(2):412–422. 10.21037/apm-21-2161 34775775

[94] Sudore RL, Lum HD, You JJ, Hanson LC, Meier DE, Pantilat SZ, et al. Defining advance care planning for adults: a consensus definition from a multidisciplinary Delphi panel. J Pain Symptom Manage 2017;53(5):821–32.e1. 28062339 10.1016/j.jpainsymman.2016.12.331 PMC5728651

[95] Detering KM, Hancock AD, Reade MC, Silvester W. The impact of advance care planning on end of life care in elderly patients: randomised controlled trial. BMJ (Clinical Research Ed) 2010;340:c1345. 10.1136/bmj.c1345 PMC2844949 20332506

[96] Peppercorn JM, Smith TJ, Helft PR, Debono DJ, Berry SR, Wollins DS, et al. American society of clinical oncology statement: toward individualized care for patients with advanced cancer. J Clin Oncol Off J Am Soc Clin Oncol 2011;29(6):755–60. 10.1200/JCO.2010.33.1744 21263086

[97] Extermann MA, Aapro M.; Audisio, R.; Balducci, L.; Droz, J.; Steer, C.; Wildiers, H.; Zulian, G. Main priorities for the development of geriatric oncology: a worldwide expert perspective. Journal of Geriatric Oncology 2011;2(4):270–3.

[98] Song P, Tang W. The community-based integrated care system in Japan: health care and nursing care challenges posed by super-aged society. Biosci Trends 2019;13(3):279–81. 31327797 10.5582/bst.2019.01173

[99] Atagi S, Kawahara M, Yokoyama A, Okamoto H, Yamamoto N, Ohe Y, et al. Thoracic radiotherapy with or without daily low-dose carboplatin in elderly patients with non-small-cell lung cancer: a randomised, controlled, phase 3 trial by the Japan clinical oncology group (JCOG0301). Lancet Oncol 2012;13(7):671–8. 22622008 10.1016/S1470-2045(12)70139-0

[100] Okamoto I, Nokihara H, Nomura S, Niho S, Sugawara S, Horinouchi H, et al. Comparison of carboplatin plus Pemetrexed followed by maintenance Pemetrexed with docetaxel monotherapy in elderly patients with advanced nonsquamous non-small cell lung cancer: a phase 3 randomized clinical trial. JAMA Oncol 2020;6(5):e196828. 32163097 10.1001/jamaoncol.2019.6828 PMC7068674

[101] Abe T, Takeda K, Ohe Y, Kudoh S, Ichinose Y, Okamoto H, et al. Randomized phase III trial comparing weekly docetaxel plus cisplatin versus docetaxel monotherapy every 3 weeks in elderly patients with advanced non-small-cell lung cancer: the intergroup trial JCOG0803/WJOG4307L. J Clin Oncol Off J Am Soc Clin Oncol 2015;33(6):575–81. 10.1200/JCO.2014.55.8627 25584004

[102] Tsukada H, Yokoyama A, Goto K, Shinkai T, Harada M, Ando M, et al. Randomized controlled trial comparing docetaxel-cisplatin combination with weekly docetaxel alone in elderly patients with advanced non-small-cell lung cancer: Japan clinical oncology group (JCOG) 0207dagger. Jpn J Clin Oncol 2015;45(1):88–95. 25378648 10.1093/jjco/hyu176

[103] Sawaki M, Taira N, Uemura Y, Saito T, Baba S, Kobayashi K, et al. Randomized controlled trial of Trastuzumab with or without chemotherapy for HER2-positive early breast cancer in older patients. J Clin Oncol 2020;38(32):3743–3752. 10.1200/JCO.20.00184 32936713

[104] Mizutani T, Ando M, Mizusawa J, Nakamura K, Fukuda H, Tsukada H, et al. Prognostic value of lung cancer subscale in older patients with advanced non-small cell lung cancer: an integrated analysis of JCOG0207 and JCOG0803/WJOG4307L (JCOG1414A). Journal of Geriatric Oncology. 2018;9(6):583–8. 29731344 10.1016/j.jgo.2018.04.005

[105] Atagi S, Mizusawa J, Ishikura S, Takahashi T, Okamoto H, Tanaka H, et al. Chemoradiotherapy in elderly patients with non-small-cell lung cancer: long-term follow-up of a randomized trial (JCOG0301). Clin Lung Cancer 2018;19(5):e619-e27. 29887243 10.1016/j.cllc.2018.04.018

[106] Wildiers H, Mauer M, Pallis A, Hurria A, Mohile SG, Luciani A, et al. End points and trial design in geriatric oncology research: a joint European organisation for research and treatment of cancer--alliance for clinical trials in oncology--international society of geriatric oncology position article. J Clin Oncol 2013;31(29):3711–8. 24019549 10.1200/JCO.2013.49.6125

[107] Hamaker ME, Stauder R, van Munster BC. On-going clinical trials for elderly patients with a hematological malignancy: are we addressing the right end points? Annals of Oncology : Official Journal of the European Society for Medical Oncology 2014;25(3):675–81. 24458474 10.1093/annonc/mdt592 PMC4433524

[108] Le Saux O, Falandry C, Gan HK, You B, Freyer G, Péron J. Changes in the use of end points in clinical trials for elderly cancer patients over time. Annals of Oncology : Official Journal of the European Society for Medical Oncology 2017;28(10):2606–11. 28961850 10.1093/annonc/mdx354

[109] SOCIETY TJG . THE JAPAN GERIATRICS SOCIETY. https://www.jpn-geriat-soc.or.jp/en/

[110] Vollset SE, Goren E, Yuan CW, Cao J, Smith AE, Hsiao T, et al. Fertility, mortality, migration, and population scenarios for 195 countries and territories from 2017 to 2100: a forecasting analysis for the global burden of disease study. Lancet (London, England). 2020;396(10258):1285–306. 10.1016/S0140-6736(20)30677-2 PMC7561721 32679112

[111] Ikegami N. Japan: achieving UHC by regulating payment. Globalization and Health 2019;15(Suppl 1):72. 31775796 10.1186/s12992-019-0524-4 PMC6882306

[112] Kojima T, Mizukami K, Tomita N, Arai H, Ohrui T, Eto M, et al. Screening tool for older Persons' appropriate prescriptions for Japanese: report of the Japan geriatrics society working group on "guidelines for medical treatment and its safety in the elderly". Geriatr Gerontol Int 2016;16(9):983–1001. 27594406 10.1111/ggi.12890

